# RNA Sequencing of Sperm from Healthy Cattle and Horses Reveals the Presence of a Large Bacterial Population

**DOI:** 10.3390/cimb46090620

**Published:** 2024-09-19

**Authors:** Paula Navarrete-López, Victoria Asselstine, María Maroto, Marta Lombó, Ángela Cánovas, Alfonso Gutiérrez-Adán

**Affiliations:** 1 Department of Animal Reproduction, INIA-CSIC, 28040 Madrid, Spain; paula.navarrete@inia.csic.es (P.N.-L.); maria.maroto@inia.csic.es (M.M.); mloma@unileon.es (M.L.); 2 Centre for Genetic Improvement of Livestock, Department of Animal Biosciences, University of Guelph, Guelph, ON N1G 2W1, Canada; vasselst@uoguelph.ca (V.A.); acanovas@uoguelph.ca (Á.C.)

**Keywords:** spermatozoa, transcriptomics, bacterial taxa, cattle, horse

## Abstract

RNA molecules within ejaculated sperm can be characterized through whole-transcriptome sequencing, enabling the identification of pivotal transcripts that may influence reproductive success. However, the profiling of sperm transcriptomes through next-generation sequencing has several limitations impairing the identification of functional transcripts. In this study, we explored the nature of the RNA sequences present in the sperm transcriptome of two livestock species, cattle and horses, using RNA sequencing (RNA-seq) technology. Through processing of transcriptomic data derived from bovine and equine sperm cell preparations, low mapping rates to the reference genomes were observed, mainly attributed to the presence of ribosomal RNA and bacteria in sperm samples, which led to a reduced sequencing depth of RNAs of interest. To explore the presence of bacteria, we aligned the unmapped reads to a complete database of bacterial genomes and identified bacteria-associated transcripts which were characterized. This analysis examines the limitations associated with sperm transcriptome profiling by reporting the nature of the RNA sequences among which bacterial RNA was found. These findings can aid researchers in understanding spermatozoal RNA-seq data and pave the way for the identification of molecular markers of sperm performance.

## 1. Introduction

Spermatozoa are highly specialized transcriptionally quiescent cells that house a diverse array of RNA molecules, some of which have been attributed to crucial functions related to fertilization, embryonic development, pregnancy outcome, and epigenetic inheritance [1,2,3]. While mature mammalian sperm contain complex populations of regulatory and functional RNAs, housekeeping RNA types, including ribosomal RNAs (rRNAs), undergo degradation, thereby halting translation at the culmination of spermiogenesis [4]. Certain sperm transcripts are remnants of the spermatogenesis, whereas others are purposefully retained and delivered to the oocyte during fertilization, possibly participating in post-testicular events. The detection of functional transcripts with pivotal roles poses a challenging task that garners the increasing attention of researchers.

High-throughput molecular tools are essential for the characterization of sperm RNAs, including the identification of transcripts involved in reproductive processes beyond spermatogenesis. Next-generation sequencing tools have been used to profile the sperm transcriptome in several mammalian species, including humans [5,6,7], cows [8,9,10], pigs [11,12], sheep [13], and horses [14], among others. However, sperm RNA has several characteristics that hinder various stages of transcriptomics analysis using RNA sequencing (RNA-seq) technology, including RNA extraction, library preparation, and bioinformatics data processing. These features include a low abundance of transcripts, the presence of non-sperm cells in the ejaculate, and the highly fragmented nature of the RNAs present. While several studies have tackled the problems associated with extraction and library preparation [5,15], certain bioinformatics issues remain unresolved. Accordingly, some authors have suggested that the presence of microbial-associated RNAs in the transcriptome may interfere with the host RNAs detected [11,16]. Understanding the intricacies of sperm transcriptome data and the problems that may arise during their analysis is key to advancing in this area of research.

Despite advances in artificial insemination and genetic selection and the enhanced management and feeding, reproductive failure remains one of the most significant problems in the dairy and beef cattle industries [17]. In addition, poor fertility of breeding stallions is a concern in the equine industry as they are not typically selected for their reproductive potential, and a considerable number of them do not pass the breeding soundness tests. New findings from studies involving human and vertebrate animal models indicate the significance of microbiomes in the urogenital tracts of both females and males for reproductive health and fertility. Consequently, the active exploration of manipulating these microbiomes to enhance reproductive efficiency has gained momentum in recent research [18]. The presence of bacteria or other microorganisms in sperm samples subjected to RNA-Seq analysis has not been widely investigated. Bacteria reach the ejaculate after passing through the male reproductive tract. Bacteria can influence male reproductive health, and some studies have effectively associated specific bacteria with semen quality parameters; for example, *Anaerococcus* and *Prevotella* were reported as potential markers for low fertility or infertility [19,20,21]. Using 16S rRNA gene sequencing, it has been suggested that bovine semen harbors a rich and complex microbiota that changes over time and during the breeding season [18]. Many of these bacteria are non-pathogenic, some can even have a beneficial effect on sperm performance, and pathogenic species may also be found [18,19,22]. The pathogenic bacteria in semen may contribute to male subfertility due to reduced sperm motility, DNA stability, and membrane integrity [18,23,24]. Besides discovering sperm-borne RNA molecules for use as molecular markers of optimal male fertility, scrutinizing the bacterial transcriptome to identify the activity of bacterial species associated with sperm quality holds promise.

The present study describes the challenges encountered during the processing of whole-transcriptome sequencing data derived from sperm samples of two livestock species, cattle and horses. After obtaining 150 paired-end RNA-seq data from bull and stallion sperm, we observed a substantial proportion of reads that did not map to the reference genome of the host species. To gain insight into the nature of the data, we examined the taxonomic profiles of these unmapped RNA transcripts and identified bacterial species contents of sperm preparations from both species. Overall, the purpose of the study is to characterize the nature of the RNA sequences derived from bovine and equine sperm cell preparations focusing on the problems associated with sperm RNA profiling while reporting the bacterial taxa found.

## 2. Materials and Methods

### 2.1. Semen Collection

Fresh semen samples were collected from four fertile purebred Andalusian stallions aged 5 to 10 years which were generously provided by the Faculty of Veterinary Sciences, Complutense University, Madrid, Spain, using an artificial vagina (Hannover model, Minitüb, Landshut, Germany). A nylon in-line filter (Animal Reproduction Systems, Chino, CA, USA) was used to eliminate the gel fraction. The sperm-rich fraction was diluted 1:2 (*v*:*v*) in INRA96 medium (IMV, L’Aigle, France) [25]. On the other hand, frozen/thawed semen samples were obtained from four Asturian Valley bulls of proven fertility aged 2–6 years using an artificial vagina, courtesy of the Regional Service of Agrifood Research and Development (SERIDA), Gijón, Spain. Details of the frozen protocol are described in [26]. All 4 stallions and 4 bulls were evaluated for health and fertility in their respective farms. Moreover, all animals were used in breeding programs following routine sperm evaluations that confirmed their good quality. In addition, upon collection, semen was initially evaluated for the following variables by subjective assessment: volume, sperm concentration, sperm morphology, and sperm motility [26]. All experimental procedures were performed according to institutional and European regulations.

### 2.2. Sample Processing

Frozen bull semen samples were thawed in a water bath at 37 °C for one minute. All aliquots were prepared under sterile conditions in a laminar flow hood to prevent contamination. Moreover, the mediums used were composed of antibiotics for controlling the bacterial contamination and growth. The extender medium consisted of tris base (hydroxymethyl-aminomethane; 2.4 g, *w*/*v*), citric acid (1 g, *w*/*v*), fructose (1 g, *w*/*v*), glycerol (7 mL *v*/*v*), (25 mg) gentamicin, 50,000 IU penicillin, and streptomycin 300 µg/mL in 100 mL of distilled water [27]. Subsequently, the fresh stallion and thawed bull semen samples were purified using an Equipure^TM^ or BoviPure^TM^ colloid density gradient, respectively (Nidacon Laboratories AB, Gothenburg, Sweden), by centrifugation onto a gradient composed of 1 mL of 40% colloid and 1 mL of 80% colloid for 10 min at 280 g. The resulting sperm pellets were isolated, washed with 3 mL of Equiwash for stallion sperm and Bobiwash for bull sperm, and subjected to centrifugation at 280 g for 5 min. Pellet volumes were measured, and both sperm motility and concentration were determined. We then analyzed sperm samples under the microscope to confirm their purity, detected possible somatic cell contamination, and confirmed that sperm with cytoplasmic droplets were also efficiently removed in the purification process. Sperm motility was assessed by introducing 6 μL of a sperm suspension of each sample in a Makler^®^ chamber on the stage of a microscope pre-heated to 37 °C (Nickon Eclipse E400, Microscope Central, Feasterville-Trevose, Pennsylvania, PA, USA) and fitted with a digital camera (Basler acA1300-200uc, Basler the power of sight, Exton, PA, USA). Five videos of 1.5 s each were recorded and analyzed using the Integrated Semen Analysis System (ISAS^®^ 2008). The parameters analyzed were as described by Pérez-Cerezales et al. [28]: straight-line velocity (VSL); curvilinear velocity (VCL); average path velocity (VAP); linearity (LIN); straightness (STR); wobble (WOB); amplitude of lateral head displacement (ALH); and beatcross frequency (BCF). Sperm concentrations (sperm cells/mL × 10^6^) were determined in a Thoma^®^ counting chamber, and the resultant volume (mL) from each gradient was measured before snap-freezing in liquid nitrogen for later RNA isolation. Finally, the sperm pellets were cryopreserved in liquid nitrogen and stored at –80 °C for subsequent RNA extraction.

### 2.3. RNA Extraction

In all samples, total RNA was isolated using a TRIzol^®^ RNA reagent and extraction method following the manufacturer’s recommended protocols (Invitrogen, Carlsbad, CA, USA) [29]. Extracted RNA was then subjected to DNAse treatment (Promega, Fitchburg, WI, USA) for 15 min and further purified by phenol:chloroform extraction [30]. The resultant purified total RNA was stored in nuclease-free water. RNA concentration was determined using a spectrophotometer [31], and RNA quality was assessed in an Agilent 2100 bioanalyzer system (Agilent, Santa Clara, CA, USA) using the Agilent Small RNA Kit.

### 2.4. RNA Sequencing Analysis

Total RNA-seq analysis was conducted on sperm samples from the eight animals. Briefly, ribosomal RNA was removed from total RNA using the NEBNext^®^ rRNA depletion kit (New England Biolabs, Beijing, China), and subsequently, ethanol precipitation and fragmentation were performed to produce RNA fragments of a suitable size for sequencing. Subsequently, directional libraries were constructed using the NEBNext^®^ Ultra^TM^ RNA Library Prep Kit (New England Biolabs, Beijing, China), which employs the dUTP method to ensure strand specificity. The resultant cDNA libraries were employed for sequencing on an Illumina NovaSeq 6000 sequencer (Illumina, San Diego, CA, USA), generating 150 bp paired-end reads per sample.

Trimming and quality control procedures were conducted using the CLC Genomics Workbench (CLC Bio Version 20.0.4, Aarhus, Denmark) [32,33]. First, trimming encompassed the removal of Illumina adapter sequences, low-quality sequences (limit error probabilities = 0.05), and ambiguous nucleotides, with a maximum of 2 nucleotides allowed. After the reads were trimmed, quality control was performed using the NGS quality control tool of CLC Genomics Workbench, taking into account 50% GC content; 100% coverage in all bases; 25% of A, T, G, and C nucleotide contributions; and less than 1% over-represented sequences, as described by Cánovas et al. [34]. To assess the presence of fragmented rRNA, reads were aligned with bovine and equine rRNA sequences (including 5S, 5.8S, 18S, 28S) accordingly using Bowtie 2 v 2.4.4 [35]. The reads that mapped to rRNA were excluded from further analysis.

Next, the processed sequences from both the bovine and equine samples were aligned with their respective reference genomes: *Bos Taurus* ARS-UCD1.2 reference genome for bulls and *Equus Caballus* EquCab3.0 reference genome for stallions using the “RNA-seq analysis” algorithm implemented in the CLC Genomics Workbench [36,37]. The following default parameters were used: match score = 1; mismatch cost = 2; insertion and deletion cost = 3; length fraction = 0.8; and similarity fraction = 0.8. The high proportions of unmapped paired-end reads were then retrieved for subsequent analysis using the “Create list of unmapped reads” option.

### 2.5. Bacterial RNA Characterization

Reads which were not aligned to the host genome were examined for the presence of bacterial RNA by employing Kraken2 v 2.1.2 [38]. Initially, a custom database was generated with the code kraken2-build --build after downloading the RefSeq complete bacterial genome assemblies by using --download-taxonomy and --download-library bacteria. The unmapped paired-end reads were then aligned with this database, leading to the taxonomic assignment of the reads. Specifically, reads were assigned to the lowest common ancestor according to a confidence score exceeding 0.2 to improve the accuracy.

To accurately estimate the abundance of genus- and species-specific RNA within the sperm samples, Kraken2 classification report files were used as input for Bracken [39]. First, using Kraken2, we classified reads to multiple levels of the taxonomic tree, and next, Bracken produced accurate abundance estimates at a single level (i.e., species or genus). To execute Bracken, read counts higher than 100 were considered for a species to be re-estimated in order to exclude false positives, prioritizing the retention of the most reliable bacterial-associated reads.

## 3. Results

### 3.1. Quality Assessment of RNA-Seq Data and rRNA Filtering

**Figure 1:**
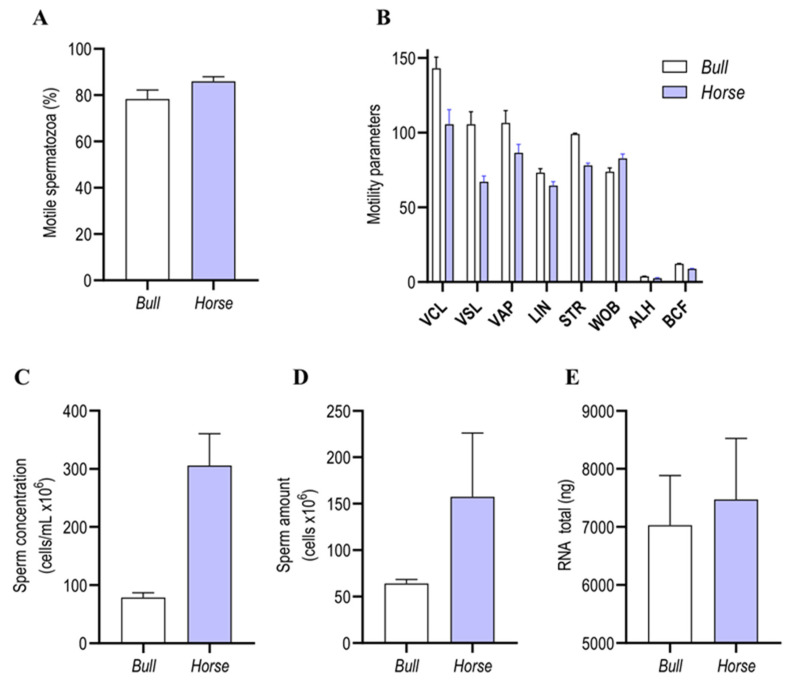
Characteristics of sperm in frozen bull and fresh stallion semen. Semen samples obtained from four bulls and four horses were processed as described in Materials and Methods. Sperm quality was assessed (**A**) by quantifying motility and (**B**) in terms of motility parameters (VCL, VSL, VAP, LIN, STR, WOB, ALH, and BCF), (**C**) sperm concentration (cells/mL × 10^6^), (**D**) sperm count (cells × 10^6^), and (**E**) total RNA. Values are expressed as mean and SEM.

Animals were selected based on good reproductive parameters and proven fertility. Additionally, we confirmed the good quality of semen samples before RNA extraction and sequencing. Mean sperm motilities were 78.28% and 86% (Figure 1A), respectively, and motility parameters (Figure 1B) and concentrations (Figure 1C) were normal. Means of 64.17 and 157.5 million bull and stallion sperm, respectively, were obtained (Figure 1D), with a minimum mean of 7 μg of total RNA for both species (Figure 1E).

**Figure 2:**
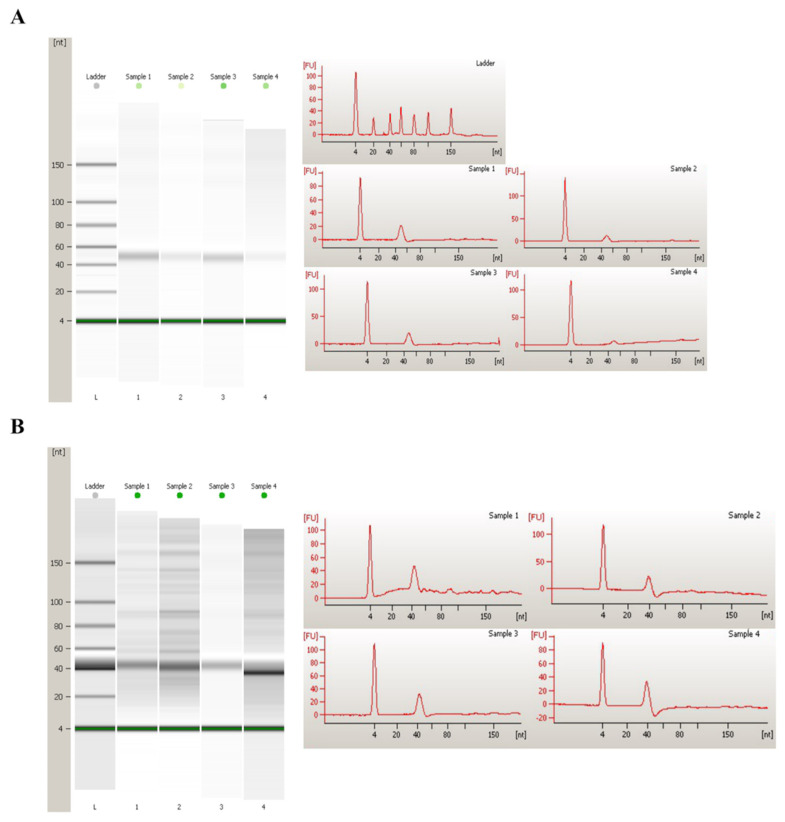
Agilent 2100 bioanalyzer gel image and electropherograms of total RNA isolated. (**A**) Frozen/thawed sperm samples from 4 bulls. (**B**) Fresh refrigerated sperm samples from 4 stallions. In the electropherogram, the chip from the Agilent Small RNA Kit shows the peak for the lower marker (4 nt), miRNAs region (4–40 nt), tRNAs region (40–80 nt), and the small RNA content (over 80 nt).

To identify transcripts associated with sperm function in the two livestock species, four libraries were constructed for each species and sequence on the Illumina platform. Prior to sequencing, the good quality of the isolated total RNA was confirmed. Bioanalyzer profiles revealed several significant points. First, the presence of partially degraded RNA was indicated by an RIN (RNA Integrity Number) value below 3 in all samples, which is characteristic of high-quality sperm. Moreover, the absence of discernible 28S and 18S peaks signified the absence of contamination by somatic cells containing intact rRNA (Figure 2). For comprehensive transcriptome coverage, we included a ribosomal RNA depletion step so that all RNA species, mRNAs, lncRNAs, and circRNAs could be analyzed.

The total number of raw reads obtained after sequencing was variable, ranging from 15 million to 26 million reads in bull sperm (Table 1) and from 67 to 106 million reads in stallion sperm (Table 2). To ensure the reliability of subsequent analyses, Illumina adapters and low-quality bases were trimmed, and low-quality reads were removed. Possible rRNA contamination was observed in the equine data, as their quality control revealed the presence of overrepresented sequences that aligned with rRNA. Despite the absence of the complete set of rRNA molecules displayed in the bioanalyzer profile and the rRNA depletion step before sequencing, we found a high percentage of reads mapped to the rRNA sequences in the equine sperm samples: 15% to 33% in the different samples (Table 2). These sequence reads were filtered out prior to the mapping step. The entire number of reads detected in horse sperm as belonging to rRNA corresponded to the 28S sequence, and specifically to two fragments of ~300–400 nucleotides. Finally, 40 to 82 million trimmed and filtered reads were used for subsequent analysis of horse data, which is a considerable reduction compared to raw reads (Table 3). As with other RNA species, rRNA is degraded in sperm, thus explaining the absence of peaks in the Bioanalyzer. Moreover, the fact that rRNA in sperm is fragmented could be the reason why rRNA is not adequately depleted.

### 3.2. Detection of Bacterial Contents

Upon aligning the reads to the host reference genomes, approximately 60% of reads in both species were successfully assigned to specific locations in the host reference genomes, thereby giving rise to low mapping rates and to around 40% of unmapped reads (Table 1 and Table 3), while usually, in RNA-seq data from biological tissues, 10% at most is expected. These high ratios of unmapped reads imply a lower sequencing depth covering the RNA types under study. To explore the notion that these unmapped reads could be associated with bacteria attached to the surface of spermatozoa, we characterized the reads that did not map to the host reference genomes of each livestock species.

Unmapped reads were aligned with the complete genomes of bacterial taxa sourced from RefSeq, and those corresponding to bacteria were accordingly identified and taxonomically classified. Unmapped paired reads were initially assigned to bacteria using Kraken2 and a confidence score exceeding 0.2, which is the number of k-mers matching the lowest common ancestor divided by the total number of k-mers. Thus, by analyzing bacterial communities through RNA-Seq, we were able to detect functional bacteria, allowing for an assessment of both their abundance and gene expression activity.

Rates of unmapped paired reads aligning to all bacterial species ranged from 13.4% to 27.2% across different individuals in bulls and from 14.1% to 20.2% in stallions. On average, bulls showed a slightly higher percentage of reads aligning with bacterial transcripts (Table 4 and Table 5).

### 3.3. Identification of Bacterial Species

To estimate abundance, we employed Bracken, retaining only those species with counts exceeding a threshold of 100 to ensure reliability. It should be noted that, by abundance, here, we refer to the abundance of the transcriptome, which does not directly correlate with the abundance of the bacteria themselves.

**Figure 3:**
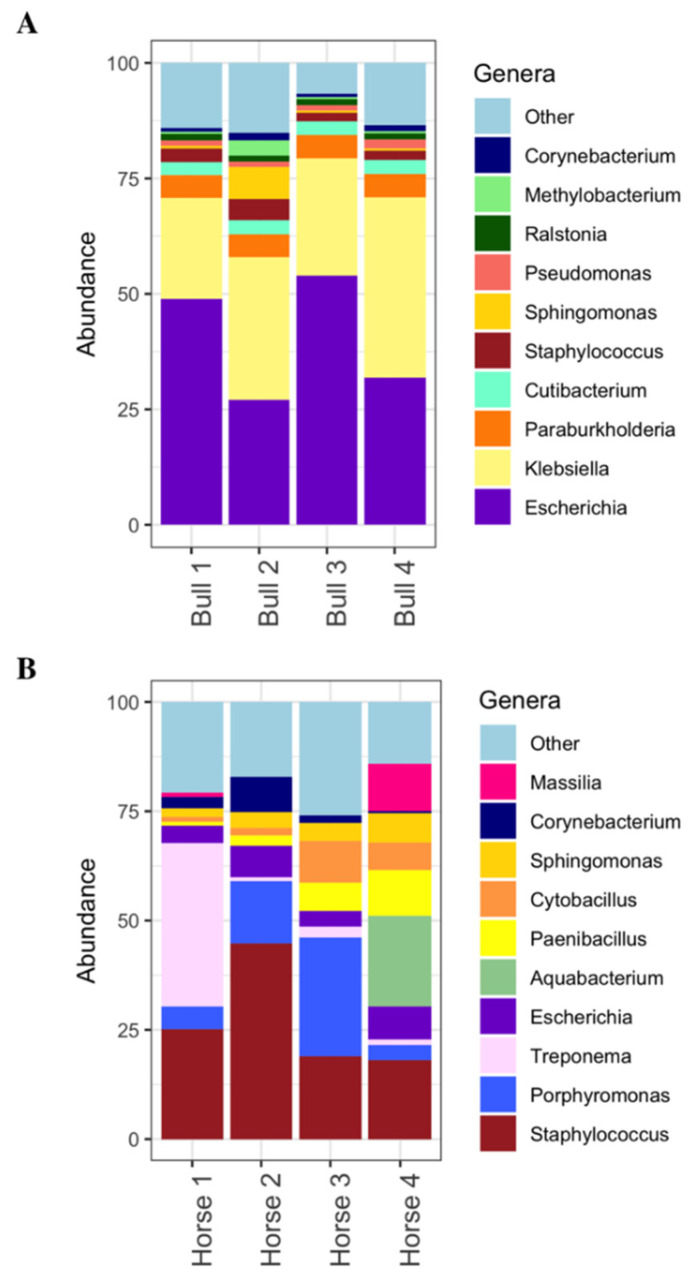
Relative abundances of bacterial genera identified in the sperm RNA obtained from (**A**) bulls and (**B**) stallions. The image shows the distributions of the top 10 most abundant genera on average for each livestock species.

In bull spermatozoa, bacterial transcriptomes corresponded to 90 genera in total, and these varied among individuals, as detailed in Table S1. Notably, *Escherichia* and *Klebsiella* were the predominant genera, significantly surpassing all others (Figure 3A). Specifically, two bulls (bull 1 and bull 3) showed higher proportions of reads assigned to *Escherichia*, accounting for roughly 50% of bacterial contents. In contrast, in the remaining two bulls (bull 2 and bull 4), *Klebsiella* was the predominant genus, accounting for over 30%.

Following *Escherichia* and *Klebsiella*, the next most abundant bacterial genera in most cows were *Paraburkholderia*, *Cutibacterium*, and *Staphylococcus*, although *Sphingomonas* held the third most abundant position in bull 2. This can be observed in Figure 3A, which depicts genus distributions in each animal. The total number of bacterial species identified in bull sperm was 153, as detailed in Table S1.

Among the species identified, several were consistent across all four bulls: *Escherichia coli*, *Klebsiella pneumoniae*, *Paraburkholderia fungorum*, *Cutibacterium acnes*, and *Staphylococcus aureus*. However, some animals featured specific bacteria among their top species, which were notably reduced in the remaining bulls. For instance, *Xanthomonas campestris* was distinctive to bull 1, *Cytobacillus oceanisedeiminis* to bull 2, and *Salmonella enterica* to bull 4.

Table S2 presents the bacterial genera and species found in stallion semen. In total, we identified 83 genera and 165 species. On average, the most prevalent bacterial genus across all horses was *Staphylococcus*. However, there was marked individual variation, with the most abundant bacteria in each horse being *Treponema*, *Staphylococcus*, *Porphyromonas*, and *Aquabacterium* (Figure 3B).

The most frequent species across the different horse sperm samples was always *S. aureus*, which appeared in proportions of 18% to 43%. Notably, species of the genus *Porphyromonas*, especially *P. cangingivalis* and *P. somerae*, were found to play important roles in horses, consistent with previous findings [40,41]. Two *Treponema* species, including *T. phagedenis*, were among the most abundant in horse 1. *T. phagedenis* has been associated with infectious diseases such as digital dermatitis in dairy cattle and hoof canker in horses [42]. *Escherichia coli* was consistently present in all horses, although it was less abundant than in bulls. Specific species, like *Cytobacyllus ocenaisediminis* and *Paenibacillus* ssp. *B01*, appeared in some of the horses. Further, several *Sphingomonas* species were found in all horses.

Notably, the most abundant species identified in the semen of one horse (horse 4) was *Aquabacterium olei*, which was absent in the other horses. Additionally, *Massilia* species were detected as one of the most abundant species. As the habitat of these species is water, soil, or plants, semen samples in horse 4 may have been exposed to environmental sources of contamination due to husbandry conditions or semen collection practices. Compared to cattle, we observed a more heterogeneous bacterial population profile in horses, perhaps because semen samples were fresh rather than frozen/thawed. In contrast, bulls featured fewer species and less variability such that the profiles were fairly similar among individuals.

## 4. Discussion

Characterizing sperm RNA is met with numerous challenges largely stemming from the different sample preparation procedures, library construction methods, and sequencing platforms used in research. Despite these varying approaches, many studies have obtained less-than-ideal results. A significant focus of research groups has been the identification of molecular markers associated with sperm performance and transgenerational inheritance. Thus, RNA-seq technology has played a crucial role in investigating the relationship between RNA species in ejaculated spermatozoa and male fertility. However, a common feature of studies examining sperm transcripts through RNA-seq has been low mapping rates to the host genome, a limitation observed in previous studies across different mammalian species [9,10,14,16]. A low mapping rate translates to reduced coverage or sequencing depth for RNA types of interest, particularly regulatory and functional RNAs. In some cases, researchers have tried to improve coverage by pooling different libraries together, as seen in cattle studies [8]. The extraction and processing procedures have been benchmarked in other studies [5,15], but the cause of the poor sequencing outcomes is yet unknown. The experimental procedure differs widely between studies, and although some offer better results than others, most of the publications report low mapping rates; thus, this issue is observed indistinctly and the rationale behind this problem has not been thoroughly explored. Our objective was to elucidate the complexity associated with the bioinformatics processing of transcriptome data derived from fresh and frozen/thawed semen samples from two different livestock species. In parallel, we examined the metatranscriptome of bovine and equine semen, shedding light on the composition of bacterial populations present in sperm.

We report the persistence of rRNA despite depletion owing to its fragmentation, as well as the presence of bacterial-associated RNAs. Sperm cells contain low amounts of RNAs, some of which are highly fragmented as a consequence of transcriptional and translational silencing [4]. This limited RNA content restricts the available library size for RNA-seq analysis. In addition to low RNA quantities, removing rRNA presents difficulties due to its degradation. Efficient rRNA depletion through hybridization is impaired by this degradation, leading to the persistence of specific RNA fragments. This issue was observed in our horse dataset, where rRNA removal resulted in a one-third reduction in sequencing depth in some samples. Consequently, it is essential that the presence of contaminating rRNA is assessed. The rRNA contamination will contribute to the low mapping rates together with the presence of bacterial RNAs. Altogether, they contribute to reducing the representation of the spermatozoal RNAs of the host, which may be of interest in transcriptomic studies, in favor of rRNA and bacterial RNA. Additionally, the remaining reads that do not correspond to rRNA or bacteria can originate from several sources: (i) novel or unannotated transcripts, which may be quite frequent in cows and horses; (ii) genomic variability: variations like single-nucleotide polymorphisms (SNPs), insertion/deletions, or structural rearrangements (e.g., translocations, inversions) can prevent proper alignment to the reference genome; (iii) sample contamination stemming from other biological contaminants (e.g., viruses) or environmental contaminants (e.g., reagents or handlers); (iv) technical errors and sequencing artifacts, such as sequencing errors; (v) degraded RNA fragments that do not match the reference; (vi) RNA editing: post-transcriptional modifications (e.g., A-to-I editing) can alter the RNA sequence.

While the most popular and cost-effective method of microbiota profiling is 16S rRNA gene amplicon sequencing, which accurately estimates bacterial abundance and captures the complete bacterial profile, we determined bacterial gene expression activity through the transcriptome, as opposed to bacterial abundance. The studies using 16S rRNA gene sequencing provide a more thorough characterization of all species present, which is not our principal objective. Therefore, and because more samples would be required, this study does not attempt to characterize the bacterial species found in semen from both species. This is a mere description of those present in the sperm transcriptomic data of the employed animals, while the main purpose is to report the limitations observed throughout the bioinformatic processing of the spermatozoal RNA-seq data of two livestock species, which will help future researchers understand and try to tackle the study of the sperm transcriptome in other animals.

Only a few studies have explored bacterial communities in sperm RNA data, particularly in human [16] and boar sperm [11]. For example, Swanson et al. (2020) assessed the human semen microbiota using transcriptome data from ejaculates and compared its profile with the results of 16S rRNA sequencing studies, thus confirming the suitability of RNA-seq for this purpose. In pigs, Godia et al. (2020) analyzed the relationship between bacterial communities and sperm quality traits, suggesting the presence of pathogens and antibiotic resistance genes that might affect boar fertility [11]. A distinctive feature of our study was the sequencing of RNA derived from purified spermatozoa where the absence of bacteria might be expected. Most studies on sperm microbiota have analyzed the complete ejaculate or seminal fluid for bacterial identification. In contrast, we were able to detect the presence of bacteria in purified sperm cells.

Other authors have relied upon 16S rRNA sequencing to characterize the microbiota present in bovine and equine semen [18,40,41,43]. In the present study, we identified a wide range of bacterial genera and species in the sperm of bulls and stallions, consistent with previous findings in both these species. There is clear evidence of high variability in bacterial profiles not only between species, but also among individuals within the same species. The semen microbiota is a dynamic environment, and the variability is attributed to geographic location, husbandry practices, breeding facility hygiene conditions, and potential contamination from feed or water sources. Not only are bacterial communities derived from the urogenital tract or preputial fluids, but also the handling protocols employed during semen collection. Because sterile conditions were used to process samples in the laboratory, the sources of the bacteria found are mainly the environment or facilities where the animals are kept, as well as the artificial vagina used during the collection step. Despite the high variability observed in previous studies, less diversity was observed in the bovine and equine transcriptomes analyzed here, with few species accounting for more than 50% of the totality of sequences. This was particularly evident in bulls, in which we consistently found *E. coli* and *K. pneumoniae* in all samples. In equine sperm, this variability was more pronounced, with each animal having two or three species predominating over the others. The reduced diversity detected could be explained by our analysis based on transcriptomes, which allowed for the detection of the most active bacteria, while most species could be less expressed. More importantly, a more diverse bacterial population is likely to be found in the seminal fluid, such that species appearing in purified sperm cells may be those strongly attached to the sperm membrane. For example, *E. coli* has been described to show an affinity for mannose receptors present in the sperm [44]. Similarly, a strong interaction between *K. pneumoniae* and sperm has been reported in humans [45].

The presence of pathogenic bacteria in semen may result in male subfertility due to reduced sperm motility, DNA stability, and membrane integrity. The two species found to show the highest relative abundance in bulls, *E. coli* and *K. pneumoniae*, are both potential pathogens that have shown negative correlations with sperm quality in some studies [21,46]. Nevertheless, in healthy bulls, sperm with better-quality traits contain a higher relative abundance of *E. coli* [18]. Therefore, the presence of *E. coli* in bovine semen appears to be beneficial, although perhaps, very high levels could be detrimental due to the agglutinating properties of this bacterial species. In the horse transcriptome, the most abundant bacterial genus represented here was *Staphylococcus*, which has been reported in human seminal fluid [19]. While culture methods have detected *Staphylococcus* as one of the most abundant bacteria in horse semen [47], sequencing methods have not identified this genus as being so relevant [41]. While *S. aureus* is not abundant in seminal fluid, it may adhere more to sperm cells. Notably, the most prevalent bacterial genus in horses is *Porphyromonas* [40,41]. Here, *Porphyromonas* was found to be the second most prevalent genus after *Staphylococcus.* In effect, as *Porphyromonas* is normally present in fertile semen, it could be a good indicator of optimal semen quality. At the same time, some pathogenic bacteria are found, such as *Salmonella* in one of the bulls, or *Treponema* in one of the horses which could indicate a urogenital infection in these two animals.

The usage of different experimental conditions, fresh and frozen/thawed samples, for each species helped to demonstrate the presence of bacteria regardless of the sample preprocessing and whether fresh or frozen/thawed samples were used. Overall, the use of two species with two different sample conditions make our work valuable, as distinct sources and conditions likewise yield poor outcomes of mapping rates that can be partly explained by the coexistence of bacteria.

## 5. Conclusions

Our research documents the presence of rRNA and bacterial-associated RNAs in purified sperm RNA-seq data despite efforts during sample processing. This hinders the study of sperm RNA associated with reproductive parameters. Concretely, this implies that the sperm transcriptome data does not only consist of spermatozoal RNA from the host, but also bacterial RNAs which, together with possible contaminating rRNA, limit the study of the RNAs of interest. These findings may provide direction for researchers seeking to examine the sperm transcriptome, offering insight into the challenges they may face.

The study highlights the occurrence of bacterial transcriptomes in purified sperm RNA-seq data while describing the active bacteria present in sperm cells in the employed animals. Regarding future directions, by profiling the bacterial transcriptome in spermatozoa, researchers may be able to assess its impact on male fertility, identifying active bacteria associated with sperm performance. Moreover, the use of purified sperm could serve to identify bacteria that may be strongly attached to the sperm surface and that may directly alter sperm’s ability to fertilize. Ultimately, the identification of bacterial species that may closely interact with sperm and potentially influence male fertility could be paramount to assessing the breeding capacity of livestock.

## Figures and Tables

**Table 1:** Raw RNA reads from bull sperm preparations mapped to the bovine reference genome using CLC genomics workbench.

Animal ID	Total Reads	Reads Mapped in Pairs		Unmapped	
		*n*	%	*n*	%
Bull 1	26,230,674	18,241,821	69.55	7,988,853	30.46
Bull 2	24,426,338	14,381,729	58.88	10,044,609	41.12
Bull 3	15,661,246	7,320,271	46.74	8,340,975	53.26
Bull 4	25,206,684	15,771,844	62.57	9,434,840	37.43
**Average**	22,881,236	13,928,916	59.44	8,952,319.25	40.57

**Table 2:** Percentage of trimmed reads mapped to horse ribosomal RNA.

Animal ID	Raw Reads	Trimmed Reads %	rRNA Reads %
Horse 1	90,408,070	3.94	18.50
Horse 2	106,877,616	4.52	27.38
Horse 3	103,473,082	4.58	15.96
Horse 4	67,010,278	8.15	33.84

**Table 3:** Trimmed RNA reads from horse sperm preparations mapping or not mapping to the equine reference genome using CLC genomics workbench.

Animal ID.	Total Reads	Reads Mapped		Unmapped	
		*n*	%	*n*	%
Horse 1	70,778,436	50,683,487	71.61	20,094,949	28.39
Horse 2	74,106,304	43,324,323	58.46	30,781,981	41.54
Horse 3	82,977,350	34,760,932	41.89	48,216,418	58.11
Horse 4	40,722,860	28,094,406	68.99	12,628,454	31.01
**Average**	67,146,238	39,215,787	60.24	27,930,451	39.76

**Table 4:** Taxonomic classification using Kraken2 (confidence score > 0.2) and Bracken (read counts > 100) of reads unmapped to the bovine genome.

Animal ID	Total Paired Reads	Reads Belonging to Bacteria		*N* Species	*N* Genera
		*n*	%		
**Bull 1**	**6,584,544**	**1,791,440**	27.21	98	62
Bull 2	8,487,712	1,140,856	13.44	85	52
Bull 3	6,995,112	1,794,728	25.66	83	51
Bull 4	7,761,026	1,279,178	16.48	79	50
**Average**	7,457,098.50	1,501,550.50	20.70	86.25	53.75

**Table 5:** Taxonomic classification using Kraken2 (confidence score > 0.2) and Bracken (read counts > 100) of reads unmapped to the equine genome.

Animal ID	Total Paired Reads	Reads Belonging to Bacteria		*N* Species	*N* Genera
		*n*	%		
**Horse 1**	**19,670,122**	**3,974,794**	20.21	78	51
Horse 2	30,262,010	4,265,912	14.10	73	46
Horse 3	47,774,260	7,756,170	16.24	129	65
Horse 4	12,050,354	2,114,412	17.55	60	36
**Average**	27,439,187	4,527,822	17.03	85.00	49.50

## Data Availability

The RNA-sequencing datasets generated and analyzed in the current study are available in GEO under accession GSE249884.

## Supplementary Materials

The following supporting information can be downloaded at: https://www.mdpi.com/article/10.3390/cimb46090620/s1, Table S1: Percentages of bacterial genera RNA present in the purified bovine sperm preparations estimated by Bracken. The genera presented are those showing an abundance greater than 0.01%. Table S2: Percentages of bacterial genera RNA present in the purified equine sperm preparations estimated by Bracken. The genera presented are those showing an abundance greater than 0.01%.

## References

[1] Krawetz S.A. (2005). Paternal contribution: New insights and future challenges. Nat. Rev. Genet..

[2] Jodar M., Selvaraju S., Sendler E., Diamond M.P., Krawetz S.A., Network R.M. (2013). The presence, role and clinical use of spermatozoal RNAs. Hum. Reprod. Update.

[3] Ostermeier G.C., Miller D., Huntriss J.D., Diamond M.P., Krawetz S.A. (2004). Reproductive biology: Delivering spermatozoan RNA to the oocyte. Nature.

[4] Johnson G.D., Sendler E., Lalancette C., Hauser R., Diamond M.P., Krawetz S.A. (2011). Cleavage of rRNA ensures translational cessation in sperm at fertilization. Mol. Hum. Reprod..

[5] Mao S., Sendler E., Goodrich R.J., Hauser R., Krawetz S.A. (2014). A comparison of sperm RNA-seq methods. Syst. Biol. Reprod. Med..

[6] Sendler E., Johnson G.D., Mao S., Goodrich R.J., Diamond M.P., Hauser R., Krawetz S.A. (2013). Stability, delivery and functions of human sperm RNAs at fertilization. Nucleic Acids Res..

[7] Corral-Vazquez C., Blanco J., Aiese Cigliano R., Sarrate Z., Rivera-Egea R., Vidal F., Garrido N., Daub C., Anton E. (2021). The RNA content of human sperm reflects prior events in spermatogenesis and potential post-fertilization effects. Mol. Hum. Reprod..

[8] Selvaraju S., Parthipan S., Somashekar L., Kolte A.P., Krishnan Binsila B., Arangasamy A., Ravindra J.P. (2017). Occurrence and functional significance of the transcriptome in bovine (*Bos taurus*) spermatozoa. Sci. Rep..

[9] Card C.J., Krieger K.E., Kaproth M., Sartini B.L. (2017). Oligo-dT selected spermatozoal transcript profiles differ among higher and lower fertility dairy sires. Anim. Reprod. Sci..

[10] Prakash M.A., Kumaresan A., Sinha M.K., Kamaraj E., Mohanty T.K., Datta T.K., Morrell J.M. (2020). RNA-Seq analysis reveals functionally relevant coding and non-coding RNAs in crossbred bull spermatozoa. Anim. Reprod. Sci..

[11] Gòdia M., Ramayo-Caldas Y., Zingaretti L.M., Darwich L., López S., Rodríguez-Gil J.E., Yeste M., Sánchez A., Clop A. (2020). A pilot RNA-seq study in 40 pietrain ejaculates to characterize the porcine sperm microbiome. Theriogenology.

[12] Fraser L., Brym P., Pareek C.S., Mogielnicka-Brzozowska M., Paukszto Ł., Jastrzębski J.P., Wasilewska-Sakowska K., Mańkowska A., Sobiech P., Żukowski K. (2020). Transcriptome analysis of boar spermatozoa with different freezability using RNA-Seq. Theriogenology.

[13] Ureña I., González C., Ramón M., Gòdia M., Clop A., Calvo J.H., Carabaño M.J., Serrano M. (2022). Exploring the ovine sperm transcriptome by RNAseq techniques. I Effect of seasonal conditions on transcripts abundance. PLoS ONE.

[14] Das P.J., McCarthy F., Vishnoi M., Paria N., Gresham C., Li G., Kachroo P., Sudderth A.K., Teague S., Love C.C. (2013). Stallion sperm transcriptome comprises functionally coherent coding and regulatory RNAs as revealed by microarray analysis and RNA-seq. PLoS ONE.

[15] Bianchi E., Stermer A., Boekelheide K., Sigman M., Hall S.J., Reyes G., Dere E., Hwang K. (2018). High-quality human and rat spermatozoal RNA isolation for functional genomic studies. Andrology.

[16] Swanson G.M., Moskovtsev S., Librach C., Pilsner J.R., Goodrich R., Krawetz S.A. (2020). What human sperm RNA-Seq tells us about the microbiome. J. Assist. Reprod. Genet..

[17] Reese S.T., Franco G.A., Poole R.K., Hood R., Fernandez Montero L., Oliveira Filho R.V., Cooke R.F., Pohler K.G. (2020). Pregnancy loss in beef cattle: A meta-analysis. Anim. Reprod. Sci..

[18] Koziol J.H., Sheets T., Wickware C.L., Johnson T.A. (2022). Composition and diversity of the seminal microbiota in bulls and its association with semen parameters. Theriogenology.

[19] Hou D., Zhou X., Zhong X., Settles M.L., Herring J., Wang L., Abdo Z., Forney L.J., Xu C. (2013). Microbiota of the seminal fluid from healthy and infertile men. Fertil. Steril..

[20] Weng S.-L., Chiu C.-M., Lin F.-M., Huang W.-C., Liang C., Yang T., Yang T.-L., Liu C.-Y., Wu W.-Y., Chang Y.-A. (2014). Bacterial Communities in Semen from Men of Infertile Couples: Metagenomic Sequencing Reveals Relationships of Seminal Microbiota to Semen Quality. PLoS ONE.

[21] Vilvanathan S., Kandasamy B., Jayachandran A.L., Sathiyanarayanan S., Tanjore Singaravelu V., Krishnamurthy V., Elangovan V. (2016). Bacteriospermia and Its Impact on Basic Semen Parameters among Infertile Men. Interdiscip. Perspect. Infect. Dis..

[22] Givens M.D., Marley M.S. (2008). Pathogens that cause infertility of bulls or transmission via semen. Theriogenology.

[23] Villegas J., Schulz M., Soto L., Sanchez R. (2005). Bacteria induce expression of apoptosis in human spermatozoa. Apoptosis.

[24] Tvrdá E., Ďuračka M., Benko F., Lukáč N. (2022). Bacteriospermia—A formidable player in male subfertility. Open Life Sci..

[25] Ruiz-Díaz S., Oseguera-López I., De La Cuesta-Díaz D., García-López B., Serres C., Sanchez-Calabuig M.J., Gutiérrez-Adán A., Perez-Cerezales S. (2020). The Presence of D-Penicillamine during the In Vitro Capacitation of Stallion Spermatozoa Prolongs Hyperactive-Like Motility and Allows for Sperm Selection by Thermotaxis. Animals.

[26] Muiño R., Peña A.I., Rodríguez A., Tamargo C., Hidalgo C.O. (2009). Effects of cryopreservation on the motile sperm subpopulations in semen from Asturiana de los Valles bulls. Theriogenology.

[27] Santos C.S., Silva A.R. (2020). Current and alternative trends in antibacterial agents used in mammalian semen technology. Anim. Reprod..

[28] Pérez-Cerezales S., Laguna-Barraza R., de Castro A.C., Sánchez-Calabuig M.J., Cano-Oliva E., de Castro-Pita F.J., Montoro-Buils L., Pericuesta E., Fernández-González R., Gutiérrez-Adán A. (2018). Sperm selection by thermotaxis improves ICSI outcome in mice. Sci. Rep..

[29] Horiuchi K., Perez-Cerezales S., Papasaikas P., Ramos-Ibeas P., López-Cardona A.P., Laguna-Barraza R., Fonseca Balvís N., Pericuesta E., Fernández-González R., Planells B. (2018). Impaired Spermatogenesis, Muscle, and Erythrocyte Function in U12 Intron Splicing-Defective Zrsr1 Mutant Mice. Cell Rep..

[30] Gómez-Redondo I., Ramos-Ibeas P., Pericuesta E., Fernández-González R., Laguna-Barraza R., Gutiérrez-Adán A. (2020). Minor Splicing Factors Zrsr1 and Zrsr2 are essential for early embryo development and 2-cell-like conversion. Int. J. Mol. Sci..

[31] Michailidou K., Lindström S., Dennis J., Beesley J., Hui S., Kar S., Lemaçon A., Soucy P., Glubb D., Rostamianfar A. (2017). Association analysis identifies 65 new breast cancer risk loci. Nature.

[32] Cánovas A., Reverter A., DeAtley K.L., Ashley R.L., Colgrave M.L., Fortes M.R., Islas-Trejo A., Lehnert S., Porto-Neto L., Rincón G. (2014). Multi-tissue omics analyses reveal molecular regulatory networks for puberty in composite beef cattle. PLoS ONE.

[33] Fortes M.R., Nguyen L.T., Weller M.M., Cánovas A., Islas-Trejo A., Porto-Neto L.R., Reverter A., Lehnert S.A., Boe-Hansen G.B., Thomas M.G. (2016). Transcriptome analyses identify five transcription factors differentially expressed in the hypothalamus of post- versus prepubertal Brahman heifers. J. Anim. Sci..

[34] Cánovas A., Rincón G., Bevilacqua C., Islas-Trejo A., Brenaut P., Hovey R.C., Boutinaud M., Morgenthaler C., VanKlompenberg M.K., Martin P. (2014). Comparison of five different RNA sources to examine the lactating bovine mammary gland transcriptome using RNA-Sequencing. Sci. Rep..

[35] Langmead B., Salzberg S.L. (2012). Fast gapped-read alignment with Bowtie 2. Nat. Methods.

[36] Asselstine V., Medrano J.F., Cánovas A. (2022). Identification of novel alternative splicing associated with mastitis disease in Holstein dairy cows using large gap read mapping. BMC Genom..

[37] Klohonatz K.M., Coleman S.J., Islas-Trejo A.D., Medrano J.F., Hess A.M., Kalbfleisch T., Thomas M.G., Bouma G.J., Bruemmer J.E. (2019). Coding RNA Sequencing of Equine Endometrium during Maternal Recognition of Pregnancy. Genes.

[38] Wood D.E., Lu J., Langmead B. (2019). Improved metagenomic analysis with Kraken 2. Genome Biol..

[39] Lu J., Breitwieser F.P., Thielen P., Salzberg S.L. (2017). Bracken: Estimating species abundance in metagenomics data. PeerJ Comput. Sci..

[40] Al-Kass Z., Guo Y., Vinnere Pettersson O., Niazi A., Morrell J.M. (2020). Metagenomic analysis of bacteria in stallion semen. Anim. Reprod. Sci..

[41] Quiñones-Pérez C., Hidalgo M., Ortiz I., Crespo F., Vega-Pla J.L. (2021). Characterization of the seminal bacterial microbiome of healthy, fertile stallions using next-generation sequencing. Anim. Reprod..

[42] Marčeková P., Mad’ar M., Styková E., Kačírová J., Sondorová M., Mudroň P., Žert Z. (2021). The Presence of *Treponema* spp. in Equine Hoof Canker Biopsies and Skin Samples from Bovine Digital Dermatitis Lesions. Microorganisms.

[43] Cojkic A., Niazi A., Guo Y., Hallap T., Padrik P., Morrell J.M. (2021). Identification of Bull Semen Microbiome by 16S Sequencing and Possible Relationships with Fertility. Microorganisms.

[44] Wolff H., Panhans A., Stolz W., Meurer M. (1993). Adherence of Escherichia coli to sperm: A mannose mediated phenomenon leading to agglutination of sperm and E. coli. Fertil. Steril..

[45] Zuleta-González M.C., Zapata-Salazar M.E., Guerrero-Hurtado L.S., Puerta-Suárez J., Cardona-Maya W.D. (2019). Klebsiella pneumoniae and Streptococcus agalactiae: Passengers in the sperm travel. Arch. Esp. Urol..

[46] Marchiani S., Baccani I., Tamburrino L., Mattiuz G., Nicolò S., Bonaiuto C., Panico C., Vignozzi L., Antonelli A., Rossolini G.M. (2021). Effects of common Gram-negative pathogens causing male genitourinary-tract infections on human sperm functions. Sci. Rep..

[47] Al-Kass Z., Eriksson E., Bagge E., Wallgren M., Morrell J.M. (2020). Microbiota of semen from stallions in Sweden identified by MALDI-TOF. Vet. Anim. Sci..

